# Recruitment and Retention Strategies for Community-Based Longitudinal Studies in Diverse Urban Neighborhoods

**DOI:** 10.2196/18591

**Published:** 2021-03-24

**Authors:** Emily B Ferris, Katarzyna Wyka, Kelly R Evenson, Joan M Dorn, Lorna Thorpe, Diane Catellier, Terry T-K Huang

**Affiliations:** 1 Center for Systems and Community Design Graduate School of Public Health and Health Policy City University of New York New York, NY United States; 2 Department of Epidemiology Gillings School of Global Public Health University of North Carolina Chapel Hill, NC United States; 3 Sophie Davis Biomedical Education Program City University of New York School of Medicine New York, NY United States; 4 Department of Population Health School of Medicine New York University New York, NY United States; 5 Research Triangle Institute Research Triangle Park Durham, NC United States

**Keywords:** community-based, participant engagement, natural experiment, built environment intervention, health disparities, study adaptations

## Abstract

Longitudinal, natural experiments provide an ideal evaluation approach to better understand the impact of built environment interventions on community health outcomes, particularly health disparities. As there are many participant engagement challenges inherent in the design of large-scale community-based studies, adaptive and iterative participant engagement strategies are critical. This paper shares practical lessons learned from the Physical Activity and Redesigned Community Spaces (PARCS) study, which is an evaluation of the impact of a citywide park renovation initiative on physical activity, psychosocial health, and community well-being. The PARCS study, although ongoing, has developed several approaches to improve participant engagement: building trust with communities, adapting the study protocol to meet participants’ needs and to reflect their capacity for participation, operational flexibility, and developing tracking systems. These strategies may help researchers anticipate and respond to participant engagement challenges in community-based studies, particularly in low-income communities of color.

## Introduction

Given the projection that obesity prevalence among US adults will rise to 49% by 2030 [1] and the many health problems associated with obesity [2], obesity and physical inactivity continue to be major public health issues [3-5]. Black and Hispanic communities in the United States have higher rates of obesity and physical inactivity and are disproportionately at risk of associated, myriad health issues [6-9]. Compared with non-Hispanic White adults (37.9%), both Hispanic adults of all races (47.0%) and non-Hispanic Black adults (46.8%) have a higher prevalence of obesity [10]. Among women, increased income and educational attainment are associated with decreased obesity prevalence [11]. Fewer Hispanic adults of all races (21.3%) and non-Hispanic Black adults (20.1%) met the 2008 Federal Physical Activity Guidelines compared with non-Hispanic White adults (25.6%) [12]. Given the complex drivers fueling the obesity epidemic and the entrenched social and environmental causes of health disparities, a strategic range of interventions tailored to diverse communities is critical to effectively address obesity, physical inactivity, and the associated health disparities in the United States [13].

Existing research has found associations between obesity prevalence and physical activity behaviors and many aspects of the built environment, including land use mix, connectivity [14], access to parks, and food retail options [15]. Inequities in the built environment may contribute to socioeconomic and racial disparities in obesity and physical activity. A nationally representative cohort study found that census blocks of lower socioeconomic status and a large proportion of minoritized residents had less access to physical activity facilities, which in turn was associated with increased overweight and decreased levels of physical activity [16]. As a result of institutional racism, longstanding racial residential segregation is a driving force of this type of inequitable distribution of and access to resources, with negative impacts on socioeconomic status (SES) and health outcomes [17].

With the potential to reach a large number of people and promote sustainable behavior change, built environment interventions offer a promising approach to prevent and reduce obesity [15,18]. For example, some park renovation interventions have positively impacted residents’ physical activity behaviors [19,20]. As obesogenic built environment features are more prevalent in lower-income neighborhoods and neighborhoods with large communities of color, built environment interventions can contribute to addressing racial, ethnic, and socioeconomic disparities in prevalence of obesity and levels of physical activity [16,21-23]. However, much of the limited, existing research on built environment interventions has used cross-sectional study designs and lacks a focus on communities of color or lower SES communities, suggesting a need for more rigorous study designs to provide higher quality evidence [24] and to better understand the potential impact of built environment interventions over time, especially in lower-income neighborhoods or communities of color [15,25,26].

The limited evidence base is, in part, due to challenges in evaluating built environment interventions. The potential of built environment interventions lies in their ability to shift daily habits and behaviors [27]; however, it can take time to measure and detect the impact of these everyday behavior changes on the health of the residents. Although longitudinal studies, especially randomized controlled trials, are ideal to examine the impact of built environment changes over time, this is not always possible in the real-world context [15]. Natural experiments are an appropriate method for evaluating policy or large-scale changes such as built environment interventions [24,28] but they are often difficult to implement rigorously. Systematic reviews of naturally occurring experiments evaluating the impact of policy and built environment changes on obesity highlighted the need to provide sufficient follow-up time and collect data at multiple time points to provide a more valid measurement of potential changes [15,24].

As two of the major challenges to rigorous, longitudinal natural experiments are participant recruitment and retention [29], effective participant engagement strategies and well-run study operations are key elements to ensure the study’s success. There are many potential impediments to enrolling participants in a large-scale longitudinal study and successfully following up with them. Potential participants must be willing to commit to participating over a long period and anticipate continuing to meet the study eligibility criteria. Throughout the study, some participants may lose interest or experience study burn out, whereas others may move or change their contact information without informing the research team [30]. Additional barriers emerge in communities of color and low-income communities. Mistrust of medical professionals and fear of exploitation have been identified as challenges in recruiting participants of color in the United States [31,32]. For low-income individuals of diverse racial and ethnic identities, competing demands are a major barrier and, in general, the burden of participation is much higher for low-income individuals than for higher-income study participants [31,32]. Owing to these challenges, iterative participant engagement strategies and operational flexibility to keep participants involved become critical to the success of the study, as high attrition can increase the risk of bias and impact study validity [30,33]. The growing interest and increasing body of literature exploring effective participant engagement and retention strategies reflect this importance [31,34,35].

Given the dynamic nature of longitudinal studies, research teams’ participant engagement strategies may change frequently to adapt to participant and study needs. Research teams’ documented engagement protocols, which are created either for internal use or for institutional board review, do not always reflect these adaptations and the full range of strategies actually used by a research team [33]. A review of studies with high participant retention found that research teams iteratively adapted and tailored engagement strategies throughout the life of the study based on the specific needs of participants and that this process was rarely documented [33]. Successful participant engagement strategies are often labor-intensive, including substantial *in-the-field tracking*, and iteratively translate insights from researchers and participants into protocol adaptations [31,36]. This paper, presented as a practical viewpoint from the field, seeks to document insights learned from the Physical Activity in Redesigned Community Spaces (PARCS) study regarding adaptive participant engagement strategies in a natural experiment evaluating changes to the built environment. These insights demonstrate the diverse range of strategies and the related operational flexibility that research teams can deploy to effectively engage participants, particularly from low-income communities and/or communities of color, in longitudinal studies.

## PARCS Study Background

The PARCS study is a longitudinal, natural experiment evaluating the impact of the Community Parks Initiative, an equity-based renovation initiative by the New York City Department of Parks and Recreation (NYC Parks), on residents’ physical activity, mental health, and community well-being. The Community Parks Initiative renovations focus on neighborhood parks that had not received significant capital investment in the past two decades and met two of the following three criteria: above average population density, above average percentage of residents living below the federal poverty line, and recent population growth. Control parks were matched based on neighborhood demographic characteristics and met the Community Parks Initiative inclusion criteria but were not slated for renovation during the study timeline. The full research protocol has been published elsewhere [37]. This study was approved by the City University of New York Institutional Review Board. All participants provided informed consent before their inclusion in the study.

Baseline data were collected from June 2016 to August 2018, with the initial goal of recruiting approximately 1700 participants. For most study sites, the second wave of data collection occurred 2 years after baseline data collection and wave 3 occurred 3 years after baseline. However, due to changes in the park renovation timelines, the second wave of data collection took place 3-4 years after baseline in the selected sites. Although many studies experience more difficulty recruiting control participants compared with intervention participants, this has not been the case in the PARCS study. Although information about parks included in the Community Parks Initiative is publicly available, PARCS study participants were not explicitly told the study arm of their neighborhood site to minimize potentially influencing participants’ park use behaviors, and the preliminary average number of participants per study site at baseline was similar in the intervention and control sites (approximately 30 participants per site). In a preliminary baseline sample, 54.2% of participants reported an annual income of ≤US $20,000, and the majority were identified as Hispanic of any race (43.1%) or non-Hispanic Black (49.5%) [38]. As follow-up data collection is ongoing, retention rates are not yet known.

All participants lived within 0.3 miles from one of the 54 study parks (33 intervention parks and 21 control parks). The study included two cohorts: an adult-only cohort and a parent-child dyad cohort in which a primary caregiver and a child aged between 3 years and 8 years were enrolled together. Participants agreed to wear an accelerometer for at least 10 hours per day over a 7-day period to measure both physical activity and sedentary behavior. Over the same 7-day period, adult participants used the PARCS study app to respond to a survey with questions on a range of psychosocial and community well-being measures. Through the study app, participants also responded to brief real-time ecological momentary assessment surveys regarding park use. Using mobile geographic information system-enabled technology, the PARCS study app geofenced each study park and recorded participants’ usage of study parks. Field staff referred to as project coordinators were responsible for participant recruitment, implementing engagement strategies, distributing study materials, following up with participants, and providing a community-level interface for the study. Three project managers were responsible for overseeing field staff, coordinating site scheduling and accelerometer distribution, monitoring participant engagement strategies, and tracking adherence metrics to monitor progress toward study goals.

The PARCS study methodology is a response to the call for more rigorous study designs to generate stronger evidence regarding the relationship between built environment interventions and health behaviors and outcomes. As the included park renovations mainly occur in lower-income communities of color throughout New York City, the study further aims to generate evidence about the relationship between built environment interventions and health equity. Due to the long timeframe and a focus on low-income communities and neighborhoods where a majority of residents are people of color, the PARCS study team has encountered many of the previously reported challenges of successfully engaging participants [33]. Throughout the process, the research team attempted to respond by adapting diverse participant engagement and operational management strategies.

## Study Adaptations in Participant Engagement and Operational Flexibility

It is critical to employ both external (ie, participant and community focused) and internal (ie, within the research team) strategies to optimize participant engagement. Looking outward, it is key to have iterative, responsive strategies that directly engage with participants and support participants’ continued involvement in the project. It is equally important to have internal, project, and staff management strategies that are adaptable, clear, and synergistic. On the basis of assessing protocol changes to date and tracking the adaptations’ efficacy when feasible, the PARCS study team’s approach to participant engagement has centered on the following four dimensions: (1) building trust with communities, (2) adapting the protocol to meet participant capacity, (3) establishing operational flexibility, and (4) developing tracking systems. The PARCS study team identified these priority dimensions based on the key themes that emerged repeatedly throughout the study in discussions with the investigator team and study staff and that encapsulated the most pressing participant engagement and operations challenges. These priority dimensions also reflect previous research on the importance of developing trust with communities [31,32], having flexible operational structures to solicit feedback from staff and implement rapid protocol adjustments [31], and developing a consistent study identity [33,34]. Table 1 provides a summary of all strategies and the hypothesized impact.

**Table 1:** Summary of participant engagement strategies.

Methods and strategies				Hypothesized impact
**Dimension 1: building trust with communities**				
	**Creating added value**			
		Regular social media content (eg, Instagram, Facebook) Monthly touchpoints (birthday and holiday cards, pulse surveys, raffles, and photo contests)	Participants feel part of a community with shared values and mutual interest in contributing to their neighborhoods Participants have continued opportunities to engage and feel connected with the study community	
	**Maintaining a professional and legitimate presence**			
		Maximize project coordinator’s ability to quickly signal their association with known organizations Address verification	Participants recognize affiliated organizations and are more likely to trust staff and believe in the project’s legitimacy and mission Further demonstrated validity of study and level of commitment necessary to participate	
	**Branding**			
		Consistent branding of all materials	The PARCS^a^ study becomes increasingly familiar and trustworthy within study neighborhoods	
**Dimension 2. Adapting the protocol to meet participant capacity**				
	**Scheduled appointments versus rapid deployment**			
		Participants were screened and enrolled at the same initial meeting	Participants were more likely to successfully enroll	
	**Additional sites**			
		Four additional sites were added	Helped address recruitment challenges related to variance in density and zoning among neighborhoods	
	**Supplemental sample cohort**			
		A supplemental cohort was recruited at each follow-up wave of data collection	Helped address attrition	
**Dimension 3. Establishing operational flexibility**				
	**Switching from teams to one operating unit**			
		Operational structure shifted from three distinct units to one more centralized team	Increased operational flexibility allowed for scheduling to be more efficiently managed by one-point person	
	**Case management approach**			
		Field staff were responsible for checking in with the participants they enrolled	Developed rapport and a deeper connection between participants and the study	
	**Reporting mechanisms**			
		Establish communication channels so field staff can efficiently report issues from the field and managers can communicate protocol changes	Research can adapt quickly and efficiently as issues come up in the field	
**Dimension 4. Developing tracking systems**				
	**Tracking enrollment and retention**			
		Weekly tracking of the number of participants enrolled per hour worked by each project coordinator and of contact attempts to connect with returning participants for follow-up waves of data collection	Allowed staff to address any training or site-specific enrollment issues and provided field staff a sense of ownership and investment with the broader project goals and identify sites which needed additional support	
	**Protocol adherence**			
		Weekly tracking of survey completion, accelerometer return, and accelerometer wear adherence rates	Allowed staff to gauge level of participants’ engagement with protocol, provide individualized feedback to participants, and quickly identify any protocol adherence issues	

^a^PARCS: Physical Activity and Redesigned Community Spaces.

### Dimension 1: Building Trust With Communities

Starting with the initial approach and throughout follow-up interactions, it is critical to build trust and a respectful rapport with participants. The PARCS study team has employed the following methods to build and maintain trust in communities: (1) creating added value for participants, (2) maintaining a professional and legitimate presence, and (3) building a consistent brand.

#### Creating Added Value

Given the long-term nature of longitudinal research and changes to the built environment, there may not be an immediate benefit to study participants. To offset this, research teams can create an additional *value add* for participants. In a qualitative substudy using key informant interviews with 20 PARCS participants to understand their motivations for participating, many participants identified the importance of helping their communities and contributing to the society [39]. On the basis of these findings, the PARCS team recognized that the most compelling *value* *addition* for many participants may be the opportunity to contribute to their neighborhoods and to be part of a community of people with similar values. In this vein, the study team attempted to foster a sense of community among participants, aligned with participants’ values and motivations for participating.

The PARCS team primarily used social media strategies to develop this sense of community. The team posted content weekly on the PARCS study Instagram and Facebook accounts to build the study identity and develop an online community specifically for PARCS study participants. Through this content, we aimed to reflect the participants’ interest in meaningful connections and in contributing to their communities and to further convey the message that they found a community of people with shared values within the PARCS study.

The research project provided additional value through monthly touchpoints with study participants, including quarterly newsletters, holiday and birthday cards, pulse surveys (eg, short text-based surveys based on timely topics), raffles, and photo contests. The monthly touchpoints were designed to help participants feel connected to the project and to the larger PARCS community. Just as importantly, they were designed to involve minimal effort from the participant.

#### Maintaining a Professional and Legitimate Presence

With 54 sites across all five New York City boroughs and limited recruitment time windows (2-4 weeks per site), field staff needed recruitment materials that were easy to transport on public transit and communication strategies, which were effective for diverse audiences. Field staff needed to quickly establish a reputable professional presence in the study neighborhood. Given that residents must live within a 0.3-mile radius of a park to be eligible, this created hyperlocalized recruitment zones with different physical and social features. In some study sites, field staff were able to collaborate with local organizations within a 0.3-mile radius and recruited from these established local community fixtures. However, most study sites did not have this type of existing infrastructure within the study zone. At most sites, field staff relied on street-intercept recruitment strategies where they approached residents in key locations to describe the study. Due to the informal nature of street-intercept approaches, it is difficult to establish a professional rapport and to communicate the project’s legitimacy. Field staff reported receiving the following feedback from potential participants: wariness to share personal information with strangers, distrust that the study was affiliated with a credible institution, and skepticism regarding the accelerometers and fear of being *tracked* by the device.

To address these concerns, the study team developed strategies to maximize the field staff’s ability to quickly signal their affiliations with a known organization to legitimize the project. The research team purchased folding tables, banners, postcards, branded pens, and wristbands. When appropriate, the field staff set up the table and decorated it with promotional materials. Being able to approach community residents from an established (if temporary) space helped community residents feel comfortable that the study was a legitimate undertaking. Field staff were supplied with branded PARCS study t-shirts, tote bags, and lanyards to demonstrate their affiliations with a well-known official organization and project. Where it was not appropriate to use a table, field staff approached potential participants with study flyers or postcards visible so that potential participants could immediately see supporting documentation.

Upon the recommendation of a staff member with ties to some of the study communities, an optional address verification question was added to the eligibility screener. Potential participants were asked to verify their addresses with an ID or mail. This was optional, and upon meeting the rest of the eligibility criteria, participants could enroll without verifying their address. The idea was to further demonstrate the validity of the project by confirming this information. Many services that people in New York are familiar with, such as Citi Bike rentals or library services, require proof of residency. After adding this question, 714 out of 1590 screened potential participants and 551 out of 996 enrolled participants verified their addresses. The enrollment rate increased from 48% to 54% after the inclusion of the address verification question. However, address verification did not appear to have increased survey completion rates, as survey completion was similar between participants who did and did not verify their address.

#### Branding

Consistent branding is another key component of building trust and establishing the project’s legitimacy. NYC Parks created a study logo that was used on all printed materials and throughout the study’s social media presence. Many of the printed materials incorporated the City University of New York School of Public Health and NYC Parks logos to communicate the study’s affiliations to these two reputable New York City institutions. Branded promotional materials were widely distributed in the hope that the study brand would also become familiar and trusted within the community. Field staff hung large banners with study branding on study parks’ fences and distributed branded, informational materials in community centers, New York City Housing Authority (NYCHA) lobbies and tenant association offices, bodegas, laundromats, schools, day care, and libraries near study sites.

### Dimension 2: Adapting the Protocol to Meet Participant Capacity

The second critical dimension to optimize participant engagement was the study’s commitment to adapt the proposed protocol to meet participants’ capacity to participate. The PARCS study made protocol adaptations as needed in the following areas: (1) scheduled appointments versus rapid deployments, (2) additional sites, and 3) supplemental sample cohort.

#### Scheduled Appointments Versus Rapid Deployment

The initial study protocol required field staff to screen potential participants to determine eligibility and schedule follow-up appointments. At the follow-up appointment, participants would enroll in the study, receive the accelerometer, and complete the baseline survey in person with the project coordinator. The in-person appointment took approximately 90 minutes. The field staff attempted to maximize participant attendance at the scheduled follow-up appointment by meeting at the time and location of the participants’ choice. Field staff also reached out to potential participants before their appointment for confirmation. However, the rate of follow-up meeting attendance was low and would have made it impossible to meet the study recruitment targets on time. As increasing incentives and appointment reminders did not increase appointment attendance, a more substantial protocol change was required. Instead of scheduling follow-up appointments, field staff implemented a *rapid deployment* strategy where residents who met the eligibility criteria were enrolled and received all the study materials at the initial meeting. This strategy boosted recruitment numbers but, as expected, also negatively impacted survey completion adherence, as participants had the option to complete the survey at home without a project coordinator. To mitigate this negative impact, additional outreach, such as phone calls, text messages, and home visits, was added to the protocol.

#### Additional Sites

Despite adaptations to recruitment protocols, it was still difficult to achieve recruitment targets at some sites. The inclusion criteria (especially the residency requirement of living within a 0.3-mile radius of the study site and having a child between the ages of 3 years and 8 years for the parent-child dyad cohort) limited potential participants to a narrow pool. The sites also varied in terms of population density and zoning. Some study sites primarily included high-density, public housing apartment buildings, whereas others primarily included lower-density single-family residences. Zoning also differed by neighborhood. Some study sites were primarily residential, whereas others included commercial or industrial buildings, limiting recruitment potential. In other sites, geographic features (highways, rivers, etc) limited the number of residential buildings within the recruitment zone. Owing to these recruitment limitations and the associated low enrollment numbers at a few sites and after consultation with the study statistician, four additional sites (three intervention sites and one control site) were added to bolster the study sample size.

#### Supplemental Sample Cohort

As we anticipated that some participants would move outside of the study zones throughout the life of the study, we specifically recruited NYCHA residences because it is difficult to obtain an NYCHA apartment, and the buildings typically have a low turnover rate. We also only enrolled participants who said they were likely to live in the same residence for the next 4 years. Despite these precautions and accounting for some attrition due to moving, a larger number of participants than expected moved. Between waves 1 and 2 of the study, 183 participants in the adult-only cohort (16% of the cohort at baseline) moved out of the study zone and were no longer eligible to participate.

To maintain the sample size recruited at baseline in subsequent waves, we adapted the protocol to recruit supplemental sample cohorts at each follow-up wave of data collection. The study biostatistician assisted with this decision to develop an appropriate analysis plan. Supplemental sample participants were required to meet the same eligibility criteria as baseline participants. For wave 2, 28% of the adult-only cohort included supplemental sample participants.

### Dimension 3: Establishing Operational Flexibility

For the PARCS study, the third key dimension to participant engagement was operational flexibility and the ability to quickly pivot as a team. Field staff needed channels to share relevant information from the field with the management staff who had less direct contact with the participants. In turn, management staff needed flexibility to transform the field teams’ insights into protocol adaptations. Furthermore, management staff needed to communicate protocol adaptations to field staff efficiently and be confident that the adaptations would be systematically implemented. The PARCS study sought to increase operational flexibility through (1) switching from teams to one operating unit, (2) case management approach, and (3) reporting mechanisms.

#### Switch From Teams to One Operating Unit

Initially, the field staff were divided into three teams, each led by a project manager. Each team was responsible for recruitment, follow-up appointments, and participant follow-up for one site at a time. The 3-team structure made it easier to have small group meetings to discuss site-specific insights and challenges and to share site-specific materials. However, having three site-specific teams was ultimately not flexible enough to meet recruitment needs. With small, site-specific teams, it was not always feasible to schedule appointments with field staff from a specific team at study participants’ preferred time and location.

As a result, the research team restructured into one operating unit. This gave project managers more flexibility in recruitment coverage, which, in turn, made it easier to consistently schedule appointments with participants at their preferred time and location and to have sufficient staffing coverage at community events. Having one operating unit increased operational flexibility and made it easier to accommodate last-minute scheduling needs, such as attending a tenant association and meeting or picking up a participant’s accelerometer. A centralized approach allowed one project manager to handle scheduling, consistently addressing all scheduling needs and ensuring adequate coverage across all active sites.

#### Case Management Approach

Another operational challenge was discerning the best way to check in with study participants during their active data collection period and afterward if the materials were not returned. Initially, follow-up calls were made by a project coordinator scheduled to make calls. This approach, however, was unwieldy and did not strengthen the rapport with participants. If a project coordinator did not take meticulous notes, it was easy for a different project coordinator to share redundant or irrelevant information at the next call. If participants needed any follow-up or special attention, it was easy for this information to get lost.

After receiving information about these inefficiencies from the field staff, the staff switched to a case management approach. As part of the switch to case management, when field staff enrolled a new participant, they would indicate which one of them would be the *case manager* and be responsible for participant follow-up. The case manager was then responsible for checking in with the participant 2-3 times over the course of the participant’s 7-day active period via text messaging and/or phone calls. The *case manager* was responsible for logging contact attempts and taking notes. By reaching out several times throughout the active data collection period, field staff could remind participants to follow the data collection protocol, answer questions, and troubleshoot any issues. This helped build rapport between the participant and the specific field staff member who enrolled them and thus had a face-to-face connection.

#### Reporting Mechanisms

By design, there are many external factors that can impact a natural experiment but over which the research team has no control. This makes it critical to establish mechanisms for field staff to report issues as they emerge to project managers. This allows project managers to make protocol adjustments to address the problem and efficiently communicate these changes to the field staff.

For example, in the PARCS study, field staff’s early identification of mailing issues was critical for making effective protocol adjustments. As part of the wave 2 data collection protocol, field staff called returning study participants to conduct a brief rescreening survey and explain the wave 2 study protocol. All study materials were subsequently mailed to participants with a preaddressed, stamped return envelope, so that participants could mail back the accelerometer. Some participants reported issues with reliably receiving packages. Other participants reported having difficulty fitting the return envelope with the accelerometer inside their local United States Postal Service (USPS) mailboxes. Upon discovering the latter issue, the team learned that the USPS replaced all the street mailboxes in New York City with a model with a thin slit opening instead of the pull-down drawer in response to rising mailbox theft [40]. The team tried to troubleshoot this with different mailing materials, but the accelerometer dimensions were impossible to fit through the new mailboxes.

In response to these concerns, we added questions to the initial phone survey. First, the participants were asked if they could reliably receive packages at home. If not, participants could provide an alternate address or schedule a time to receive the materials in person. Most participants (96%) reported that they could receive mail at their home address reliably. The call script was also adjusted to let participants know about the blue mailbox design change and to ask if it was still convenient for participants to mail back the materials or if they preferred the materials to be picked up by a project coordinator. As 74% of participants reported that it was still convenient for them to mail the device (often at a nearby post office or workplace), we scheduled individual pick-ups for 26% of participants who requested them.

### Dimension 4: Developing Tracking Systems

The last dimension essential to participant engagement has been setting up adaptable measurement and tracking systems. It is essential to develop and track metrics to understand whether protocol adaptations address this concern. In addition to the metrics provided in earlier sections, which tracked the impact of specific protocol adjustments in real time, the team also regularly monitored (1) enrollment and retention and (2) protocol adherence to gauge overall progress and participant engagement.

#### Tracking Enrollment and Retention

During recruitment periods, project managers tracked the number of participants enrolled per hour worked by each project coordinator on a weekly basis. This helped identify whether anyone needed extra assistance with their recruitment pitch or approach. It also helped to identify whether a site needed an innovative approach or additional resources. A target number of participants to recruit per hour was established, and the staff received monetary incentive bonuses to exceed this target. Enrollment numbers were shared on a biweekly basis to provide frequent performance feedback, help field staff feel a sense of ownership in the project, and provide context for how their work supported the broader project goals.

Field staff used many approaches to connect with returning participants during follow-up waves of data collection, including phone calls, text messages, emails, letters, and flyers. Every contact attempt was tracked. Although it took an average of four contact attempts to connect with returning baseline participants for the second wave of data collection, the number of contact attempts before connecting ranged from 1 to 30. Tracking participant retention on a weekly basis helped project managers identify the sites to prioritize re-enrollment efforts and supplemental sample recruitment.

#### Protocol Adherence

Participants were asked to complete an annual survey using the PARCS study app. During the participants’ 7-day data collection period, project managers monitored participants’ survey completion progress daily through the app’s data management interface. This allowed field staff to provide individualized feedback for participants regarding their survey progress and gauge the extent of participant engagement with the study.

After a 7-day study period ended, the participant could no longer access or take the survey via the app. Owing to this and lower-than-anticipated survey completion rates, the research team mailed a paper version of the survey with a dollar bill (as added incentive) to participants who had not yet completed it. In a pilot of this protocol adaptation, 31% of adult participants at baseline who received a paper version of the survey in the mail, completed and mailed it back to us. This demonstrates that protocol adaptation is a useful and worthwhile supporting strategy to maximize survey completion.

Project managers also tracked accelerometer wear adherence and return rates on a weekly basis to further measure participant engagement.

## Conclusions

Effective participant engagement strategies are key components of the rigorous study designs needed to further develop the evidence base and better understand the health impacts of built environment interventions. Effective participant engagement practices benefit from strategic adaptations and a research team’s ability to communicate, pivot, and iterate. The PARCS study, although ongoing, has centered its participation engagement strategies on the following four dimensions: (1) building trust with communities, (2) adapting the study protocol to meet participants’ needs and to reflect their capacity for participation, (3) operational flexibility, and (4) developing tracking systems.

The PARCS study’s experience with participant engagement corroborates the best practices from other studies [30-35]. Mistrust and competing priorities emerged as barriers to participation. The study team has frequently adapted and experimented with different engagement strategies to address these barriers. In addition, deploying multiple synergistic strategies is critical to meeting participants’ varied needs throughout the course of the study. Careful documentation and tracking systems, where possible, have helped identify engagement problems as they arise and determine the utility of engagement strategies.

Most of the PARCS study’s priority dimensions have been designed to specifically address some of the documented participation barriers for low-income neighborhoods and communities of color. As built environment interventions are hypothesized to have the potential to reduce socioeconomic and racial disparities in obesity outcomes and physical activity behaviors, it is critical for studies to include diverse participants to build a relevant evidence base.

A limitation of this paper is that although the research team attempted to track the impact of engagement strategies when possible, the tracking systems were added after the start of the study and were not necessarily designed to formally and empirically test the strategies. Future research studies could consider developing tracking systems *a priori* so that strategies for participant engagement and operational flexibility could be more rigorously tested. This is a potential area for future research.

This paper shares practical lessons about iterative, dynamic strategies to improve participant engagement and operational flexibility based on the experience to date in an ongoing longitudinal study in low-income communities and communities of color. Insights learned from the PARCS study may help other research teams effectively anticipate and respond to participant engagement challenges in future community-based studies.

## Abbreviations

NYC Parks: New York City Department of Parks and Recreation
NYCHA: New York City Housing Authority
PARCS: Physical Activity and Redesigned Community Spaces
SES: socioeconomic status
USPS: United States Postal Service

## References

[1] Ward ZJ, Bleich SN, Cradock AL, Barrett JL, Giles CM, Flax C, Long MW, Gortmaker SL (2019). Projected U.S. State-Level Prevalence of Adult Obesity and Severe Obesity. N Engl J Med.

[2] Centers for Disease Control and Prevention (2020). The Health Effects of Overweight and Obesity. The Health Effects of Overweight and Obesity.

[3] Ogden CL, Carroll MD, Kit BK, Flegal KM (2014). Prevalence of childhood and adult obesity in the United States, 2011-2012. JAMA.

[4] Flegal KM, Kruszon-Moran D, Carroll MD, Fryar CD, Ogden CL (2016). Trends in Obesity Among Adults in the United States, 2005 to 2014. JAMA.

[5] Skinner AC, Skelton JA (2014). Prevalence and trends in obesity and severe obesity among children in the United States, 1999-2012. JAMA Pediatr.

[6] Barrington D, Baquero M, Borrell L, Crawford Natalie D (2010). Racial/ethnic disparities in obesity among US-born and foreign-born adults by sex and education. Obesity (Silver Spring).

[7] Cossrow N, Falkner Bonita (2004). Race/ethnic issues in obesity and obesity-related comorbidities. J Clin Endocrinol Metab.

[8] Zhang H, Rodriguez-Monguio Rosa (2012). Racial disparities in the risk of developing obesity-related diseases: a cross-sectional study. Ethn Dis.

[9] Wong RJ, Chou C, Ahmed A (2014). Long term trends and racial/ethnic disparities in the prevalence of obesity. J Community Health.

[10] Hales C, Carroll M, Fryar C, Ogden Cynthia L (2017). Prevalence of Obesity Among Adults and Youth: United States, 2015-2016. NCHS Data Brief.

[11] Ogden CL, Fakhouri TH, Carroll MD, Hales CM, Fryar CD, Li X, Freedman DS (2017). Prevalence of Obesity Among Adults, by Household Income and Education — United States, 2011–2014. MMWR Morb Mortal Wkly Rep.

[12] NCHS, National Health Interview Survey (2018). Age-sex-adjusted percentage of adults aged 18 and over who met 2008 federal physical activity guidelines for aerobic activity through leisure-time aerobic activity, by race and ethnicity: United States, 2018.

[13] Huang T, Drewnosksi A, Kumanyika S, Glass Thomas A (2009). A systems-oriented multilevel framework for addressing obesity in the 21st century. Prev Chronic Dis.

[14] McCormack GR, Shiell A (2011). In search of causality: a systematic review of the relationship between the built environment and physical activity among adults. Int J Behav Nutr Phys Act.

[15] Mayne SL, Auchincloss AH, Michael YL (2015). Impact of policy and built environment changes on obesity-related outcomes: a systematic review of naturally occurring experiments. Obes Rev.

[16] Gordon-Larsen P, Nelson Melissa C, Page Phil, Popkin Barry M (2006). Inequality in the built environment underlies key health disparities in physical activity and obesity. Pediatrics.

[17] Williams DR, Collins C (2001). Racial residential segregation: a fundamental cause of racial disparities in health. Public Health Rep.

[18] Sallis JF, Saelens BE, Frank LD, Conway TL, Slymen DJ, Cain KL, Chapman JE, Kerr J (2009). Neighborhood built environment and income: examining multiple health outcomes. Soc Sci Med.

[19] Cohen DA, Han B, Isacoff J, Shulaker B, Williamson S, Marsh T, McKenzie TL, Weir M, Bhatia R (2015). Impact of park renovations on park use and park-based physical activity. J Phys Act Health.

[20] Cohen DA, Han B, Isacoff J, Shulaker B, Williamson S (2019). Renovations of neighbourhood parks: long-term outcomes on physical activity. J Epidemiol Community Health.

[21] Sallis JF, Slymen DJ, Conway TL, Frank LD, Saelens BE, Cain K, Chapman JE (2011). Income disparities in perceived neighborhood built and social environment attributes. Health Place.

[22] Oreskovic NM, Kuhlthau KA, Romm D, Perrin JM (2009). Built environment and weight disparities among children in high- and low-income towns. Acad Pediatr.

[23] Molina-García Javier, Queralt A, Adams MA, Conway TL, Sallis JF (2017). Neighborhood built environment and socio-economic status in relation to multiple health outcomes in adolescents. Prev Med.

[24] Tseng E, Zhang A, Shogbesan O, Gudzune KA, Wilson RF, Kharrazi H, Cheskin LJ, Bass EB, Bennett WL (2018). Effectiveness of Policies and Programs to Combat Adult Obesity: a Systematic Review. J Gen Intern Med.

[25] Ferdinand Alva O, Sen B, Rahurkar S, Engler S, Menachemi N (2012). The relationship between built environments and physical activity: a systematic review. Am J Public Health.

[26] Audrey S, Batista-Ferrer H (2015). Healthy urban environments for children and young people: A systematic review of intervention studies. Health Place.

[27] Sallis JF, Glanz K (2006). The role of built environments in physical activity, eating, and obesity in childhood. Future Child.

[28] Craig P, Cooper C, Gunnell D, Haw S, Lawson K, Macintyre S, Ogilvie D, Petticrew M, Reeves B, Sutton M, Thompson S (2012). Using natural experiments to evaluate population health interventions: new Medical Research Council guidance. J Epidemiol Community Health.

[29] Galea S, Tracy M (2007). Participation rates in epidemiologic studies. Ann Epidemiol.

[30] Hill KG, Woodward D, Woelfel T, Hawkins JD, Green S (2016). Planning for Long-Term Follow-Up: Strategies Learned from Longitudinal Studies. Prev Sci.

[31] Ejiogu N, Norbeck JH, Mason MA, Cromwell BC, Zonderman AB, Evans MK (2011). Recruitment and retention strategies for minority or poor clinical research participants: lessons from the Healthy Aging in Neighborhoods of Diversity across the Life Span study. Gerontologist.

[32] George S, Duran N, Norris K (2014). A systematic review of barriers and facilitators to minority research participation among African Americans, Latinos, Asian Americans, and Pacific Islanders. Am J Public Health.

[33] Abshire M, Dinglas VD, Cajita MIA, Eakin MN, Needham DM, Himmelfarb CD (2017). Participant retention practices in longitudinal clinical research studies with high retention rates. BMC Med Res Methodol.

[34] Robinson KA, Dinglas VD, Sukrithan V, Yalamanchilli R, Mendez-Tellez PA, Dennison-Himmelfarb C, Needham DM (2015). Updated systematic review identifies substantial number of retention strategies: using more strategies retains more study participants. J Clin Epidemiol.

[35] Brueton VC, Tierney JF, Stenning S, Meredith S, Harding S, Nazareth I, Rait G (2014). Strategies to improve retention in randomised trials: a Cochrane systematic review and meta-analysis. BMJ Open.

[36] de Jong Jeroen PJ (2016). Surveying innovation in samples of individual end consumers. European Journal of Innovation Management 2016.

[37] Huang TTK, Wyka KE, Ferris EB, Gardner J, Evenson KR, Tripathi D, Soto GM, Cato MS, Moon J, Wagner J, Dorn JM, Catellier DJ, Thorpe LE (2016). The Physical Activity and Redesigned Community Spaces (PARCS) Study: Protocol of a natural experiment to investigate the impact of citywide park redesign and renovation. BMC Public Health.

[38] Cato MS, Wyka K, Ferris EB, Evenson KR, Wen F, Dorn JM, Thorpe LE, Huang TT (2020). Correlates of accelerometry non-adherence in an economically disadvantaged minority urban adult population. J Sci Med Sport.

[39] Swierad EM, Huang TTK (2018). An Exploration of Psychosocial Pathways of Parks’ Effects on Health: A Qualitative Study. IJERPH.

[40] Angi Gonzalez (2019). USPS replacing all NYC mailboxes for new 'anti-fishing model'.

